# The effects of massage therapy on dysmenorrhea caused by endometriosis

**Published:** 2010

**Authors:** Mahboubeh Valiani, Niloofar Ghasemi, Parvin Bahadoran, Reza Heshmat

**Affiliations:** * Department of Midwifery, School of Nursing and Midwifery, Isfahan University of Medical Sciences, Isfahan, Iran; ** School of Nursing and Midwifery, Isfahan University of Medical Sciences, Isfahan, Iran; *** Vice President, International Manual Medicine College of France and affiliated to Acupuncture School of Shanghai, Shanghai, China

**Keywords:** Endometriosis, dysmenorrhea, massage therapy

## Abstract

**BACKGROUND:**

Studying women’s quality of life, we come across some harmful effects that factor such as dysmenorrhea caused by endometriosis leaves on their lives, their ability to work, their familial relations, and their self-confidence. Due to the repeated medical follow-ups and the side effects of medical therapies and endometriosis surgeries, many patients tend to use less expensive, nonmedical, and nonaggressive methods. The present study aimed to assess the effects of massage therapy, one of the aforementioned methods on endometriosis caused dysmenorrhea.

**METHODS:**

This was a semi-empirical clinical trial. Considering inclusion criteria, 23 patients suffering from endometriosis visited the Infertility Center of Isfahan, who were later confirmed by laparoscopy or laparotomy were picked as the sample through a simple method. The visual analog scale and McGill questionnaires were used once before and twice after the end of intervention for each patient. The data were analyzed using SPSS software.

**RESULTS:**

There was a statistically significant difference between the intensity of pain before the intervention started, immediately after, and also six weeks after it (p < 0.001).

**CONCLUSIONS:**

According to the results of this study and confirmations of other ones, it seems that massage therapy can be a fitting method to reduce the menstrual pain caused by endometriosis.

Women form the most important fundamentals of a society’s health and hygiene; however, they face many various crises and problems throughout their lives that can critically affect the general hygiene of the society.^1^ Endometriosis is one of the prevalent diseases highly affect women’s quality of life during childbearing ages.^2^ Eighty nine million women of reproductive ages suffer from endometriosis,^3^ but the true extent of endometriosis has remained unknown.^4^ Endometriosis can cause side effects such as severe pelvic pain, painful intercourses, abnormal uterine bleeding, painful menstruation, and decreased fertility.^5^–^7^ All this can negatively affect women’s ability to work and have familial relations, and reduce their self-confidence.^8^,^9^ In addition, it frequently brings the patients to the medical centers. One of the most common side effects of endometriosis is painful menstruations or as it is called, dysmenorrhea. The prevalence of dysmenorrhea varies from 18 to 81 percent depending on the measuring method.^10^–^11^ Endometriosis, adenomiosis, and IUD (Intra uterine device) are orderly the most common causes of secondary dysmenorrhea.^7^

French not only name the painful menstruation as one of the common problems during the childbearing ages that force women to take days off work, but also emphasizes the necessity of utilizing methods to remove the problem.^12^ Barnard defines the endometriosis pain as a much more persistent pain along with cramps or pelvic pains in the beginning or near the end.^13^ In a qualitative study by Denny concerning women suffering from endometriosis, it is stated that women have used words such as extremely severe, killing, and unbearable to describe the pain.^14^ Since there is no cure for endometriosis, the medical and surgical treatments are more considered to achieve pregnancy, reduce the symptoms, and prevent the disease progression.^15^ The retrospective findings show that the common medical and surgical treatments for endometriosis have little longterm effects in reducing the symptoms, while each one brings many definite side effects along.^9^

Wurn et al showed that massage therapy reduces uterine spasms and cervix adhesion.^16^ The results of Ebrahim zade study on 216 teenager girls suffering from dysmenorrheal also illustrated that acupressure reduces the severity of the disease.^17^ Thus, it seems that presence of a nonmedical and nonaggressive method is the appropriate preference to reduce the effects of the disease.^18^

Wurn et al also examined the effects of massage on various points of abdominal and pelvic soft tissues; they concluded that this procedure reduces the pelvic pains, in addition to increasing fertility in women who are infertile (due to adhesion and dysfunction in their reproductive organs).^16^ The results of Randine study on 18 endometriosis patients, who were later confirmed through laparoscopy or laparotomy, showed a significant decrease (p = 0.001) of pelvic pain in response to massage therapy (before but close to menstruation and ovulation).^19^

The results of some articles declare that massage therapy can be effective, but the exact extent of its effects on treating endometriosis or its reduction is not quite identified yet.^16^,^18^–^20^

Massage therapy is a safe, nonaggressive, and simple method that carries very few, yet reversible side effects.^16^,^18^

Today, the only treatment for endometriosis is surgery with costly medical procedures. Since the quality of life has positive effects on the individual’s health, mental condition, independency level, social relations, and other aspects, we analyzed massage therapy as a simple, nonaggressive method on reducing dysmenorrhea caused by endometriosis especially among those patients who did not will to go under surgery or could not afford to; or the ones who had, but were still suffering from the disease.

## Methods

This was a semi-empirical clinical trial with cross-sectional method. It was a three-step research in which 30 patients were enrolled according to inclusion criteria. They were women between the ages of 15 to 49 years suffering from dysmenorrhea and there was no other diagnosed reason for their illness but symptomatic endometriosis diagnosed via laparoscopy or laparotomy. The visual analog scale (VAS)^21^ was used to measure the severity of pain and McGill questionnaires for personal/fertility information were used to collect other data.

After reviewing the patients’ files and observing the registered diagnosis of endometriosis, they were invited to the Infertility Center to receive the complete explanation of the research. Among the participants in the first session, those who willed to take part in the study were interviewed to complete the questionnaires. This initial invitation went on several times for the 30 patients to be picked using a simple method. The researcher was not in any way biased in picking the patients, since the limited number of patients was a major factor in picking the ones who wished to participate. Thus the number of chosen sample units was more than the calculated number so that if anyone declared unwillingness to continue working and left the study, the researcher would not face a shortage in samples.

The questionnaire was completed once before the beginning and twice after the end of the intervention (once immediately after the first post-intervention menstruation and once six weeks after that). The intervention consisted of 20 twenty-minute sessions of massage on some specific points of abdomen, sides, and the back (sacrum) of patients. The researcher did the massage after a few minutes when the patient had drifted off to make her relax. To do this, hands were drenched in oil and massaged the patient’s abdomen, sides, and back.

The researcher had been trained for twenty sessions to correctly utilize the massage skills. After being approved for her massage skills (finding the specific points, massage duration, and the pressure on each point) and receiving the certificate, the researcher performed the first step during which the required observations were made to secure the process. The massage therapy technique consisted of pressuring 24 specific point of abdomen and 10 points on the sacrum each one for two minutes while hands keep moving on the abdomen, sides, and sacrum.

The Cronbach’s alpha coefficient was used to validate the questionnaire. The questionnaire was confirmed with a Cronbach’s alpha coefficient of 0.7.

The data was analyzed by descriptive and inferential statistical methods (Wilcoxon signedrank test). The VAS measured data were compared by Friedman test. The Wilcoxon signedrank test was used to compare data too. Statistical analysis was done by SPSS software.

## Results

Considering the loss of sample units due to their unwillingness to continue participating in the study the number dropped from a total 30 (which was more than the calculated number) to 23. The sample units were all of ages 21-40 years, the majority of whom were 26-30 years old and the average age was 28.2 ± 4.2 years. Most of the patients had a normal body mass index (BMI) around 22.96 ± 3.59 kg/m^2^. 69.6 percent of the patients felt dysmenorrhea after and during their menstruation and 60.9 percent of them took analgesics before the study.

The highest frequency of menstrual pain before the beginning of the intervention was that of “severe pain” (52.3 percent); while the highest frequency of menstrual pain for “no pain” was that of immediately after the intervention and six weeks after the intervention (34.8 and 65.2 percent respectively). The Friedman test showed that the severity of pain was significantly in three studied times (p < 0.001). The Wilcoxon test also showed that the severity of pain during those three times have a significant difference (p < 0.001) with each other. Also the severity of the pain before intervention had a significant difference (p < 0.001) with that after it and this difference persisted after six weeks (p < 0.001). In addition, the severity of pain immediately after the end of intervention shows a significant difference from that of after six weeks (p = 0.001). The t-test showed a significant difference between before the intervention and immediately it (t = 7.831; p < 0.001) and also between before the intervention and six weeks after (t = 9.755; p < 0.001) (Table 1).

**Table 1:** Frequency of menstrual pain score (VAS) of units under study in three different periods of the research

Medical Step	Before intervention		Immediately after the intervention		Six weeks after the intervention	
Menstrual Pain Severity	Number	Percentage	Number	Percentage	Number	Percentage
0 (No pain)	0	0	8	34.8	15	65.2
1-3 (Mild)	1	4.3	6	26	7	30.5
4-6 (Moderate)	10	43.4	5	21.7	1	4.3
7-10 (Severe)	12	52.3	4	17.3	0	0
Total	23	100	23	100	23	100

## Discussion

The findings of this research showed that the effects of the massage therapy are more at six weeks after it, when the body has adjusted itself to the new conditions. Wurn et al concerning the reduction of the pelvis pains and the pain during the intercourse stated that body needs two weeks time after therapy to adjust to the changes.^19^

In all the previous studies on the effects of massages on pelvis pains, such as the menstrual pain and the pain during intercourse, the effects of massage have been evaluated only before and six weeks after the intervention; but in this study, we evaluated this effect immediately after the intervention too, in order to be able to evaluate the speed of intervention’s effectiveness. The findings showed a significant change in the level of pain immediately after the intervention, while the effects of massage increased six weeks after the intervention and the level of analgesia reached 65.2% (six weeks after the intervention) from 34.8% (immediately after the intervention) and none of the patients has experienced severe pain after the intervention. Wurn et al studied the menstrual pain caused by endometriosis after massage during the three phases of ovulation, before menstruation, and during menstruation and observed significant reduction in the menstrual pain in all three phases (p = 0.014).^20^

Ebrahim zadeh, also examined the effects of acupressure on 216 teenage girls suffering from dysmenorrhea. He found that the severity of dysmenorrhea after the acupressure therapy decreased significantly (p < 0.001).^17^

## Conclusion

According to the findings of this study, it can be stated that massage therapy can result in reducing the menstrual pain caused by endometriosis. Massage therapy can also be used as an inexpensive therapy in medicine and an alternative to reduce the pain in patients suffering from endometriosis. It can also be considered as a complementary therapy with no side effects. Thus, by teaching these techniques to the midwifery groups we can improve women’s health and develop the skills of this profession. By improving women’s health, their positive role in the society would become clearer, their sense of capability would be strengthened, and they would be more effective in taking care of the family health.

In this study, the standard of the research was having menstrual pain regardless of the endometriosis level of the patients, it is suggested that in the future studies, endometriosis level be considered as a variable.

The authors declare no conflict of interest in this study.
